# Perception of tobacco, cannabis, and alcohol use of others is associated with one’s own use

**DOI:** 10.1186/1940-0640-8-15

**Published:** 2013-10-19

**Authors:** Nicolas Bertholet, Mohamed Faouzi, Joseph Studer, Jean-Bernard Daeppen, Gerhard Gmel

**Affiliations:** 1 Alcohol Treatment Center, Department of Community Medicine and Health, Centre Hospitalier Universitaire Vaudois, Beaumont 21b, P2, Lausanne, CHUV 1011, Switzerland; 2 Swiss Institute for the Prevention of Alcohol and Drug Problems, Lausanne, Switzerland; 3 Centre for Addiction and Mental Health, Toronto, Ontario, Canada; 4 University of the West of England, Bristol, UK

**Keywords:** Overestimation, Substance use, Perception, Alcohol, Tobacco, Cannabis

## Abstract

**Background:**

Interventions have been developed to reduce overestimations of substance use among others, especially for alcohol and among students. Nevertheless, there is a lack of knowledge on misperceptions of use for substances other than alcohol. We studied the prevalence of misperceptions of use for tobacco, cannabis, and alcohol and whether the perception of tobacco, cannabis, and alcohol use by others is associated with one’s own use.

**Methods:**

Participants (n = 5216) in a cohort study from a census of 20-year-old men (N = 11,819) estimated the prevalence of tobacco and cannabis use among peers of the same age and sex and the percentage of their peers drinking more alcohol than they did. Using the census data, we determined whether participants overestimated, accurately estimated, or underestimated substance use by others. Regression models were used to compare substance use by those who overestimated or underestimated peer substance with those who accurately estimated peer use. Other variables included in the analyses were the presence of close friends with alcohol or other drug problems and family history of substance use.

**Results:**

Tobacco use by others was overestimated by 46.1% and accurately estimated by 37.3% of participants. Cannabis use by others was overestimated by 21.8% and accurately estimated by 31.6% of participants. Alcohol use by others was overestimated by more than half (53.4%) of participants and accurately estimated by 31.0%. In multivariable models, compared with participants who accurately estimated tobacco use by others, those who overestimated it reported smoking more cigarettes per week (incidence rate ratio [IRR] [95% CI], 1.17 [range, 1.05, 1.32]). There was no difference in the number of cigarettes smoked per week between those underestimating and those accurately estimating tobacco use by others (IRR [95% CI], 0.99 [range, 0.84, 1.17]). Compared with participants accurately estimating cannabis use by others, those who overestimated it reported more days of cannabis use per month (IRR [95% CI], 1.43 [range, 1.21, 1.70]), whereas those who underestimated it reported fewer days of cannabis use per month (IRR [95% CI], 0.62 [range, 0.23, 0.75]). Compared with participants accurately estimating alcohol use by others, those who overestimated it reported consuming more drinks per week (IRR [95% CI], 1.57 [range, 1.43, 1.72]), whereas those who underestimated it reported consuming fewer drinks per week (IRR [95% CI], 0.41 [range, 0.34, 0.50]).

**Conclusions:**

Perceptions of substance use by others are associated with one’s own use. In particular, overestimating use by others is frequent among young men and is associated with one’s own greater consumption. This association is independent of the substance use environment, indicating that, even in the case of proximity to a heavy-usage group, perception of use by others may influence one’s own use. If preventive interventions are to be based on normative feedback, and their aim is to reduce overestimations of use by others, then the prevalence of overestimation indicates that they may be of benefit to roughly half the population; or, in the case of cannabis, to as few as 20%. Such interventions should take into account differing strengths of association across substances.

## Background

According to social norms theory, our perceptions and beliefs about the “normal” behavior of others influences our own behavior [1]. For example, the belief that others drink alcohol, smoke tobacco, or use cannabis heavily is expected to influence the amount a person drinks alcohol, smokes tobacco, or uses cannabis [2]. Perceptions of how much alcohol others drink have been studied mostly among students [3-11]. Overestimating, or thinking that others drink more alcohol than oneself, is fairly prevalent and has been identified as a strong predictor of one’s own alcohol use [11-14]. This misperception seems to apply to tobacco smoking [2,15], but less is known about whether it applies to other substances, such as cannabis [15,16]. Misperceptions have been observed among men and women; nevertheless, gender is a potential predictor of misperceptions of substance use by others. Evidence suggests that the magnitude of norm misperception is influenced by gender, and that men tend to overestimate norms to a lesser extent than women for alcohol and other substances [3,15].

Various interventions have been designed to address overestimations of use by others, with varying success, among college students [17-19]. These interventions aim at correcting misperceptions by providing normative feedback on prevailing alcohol and tobacco use norms. One hypothesis is that individuals with heavy use will decrease their consumption by correcting their overestimation of use by others. A review by Moreira et al. [20] provides evidence that normative feedback interventions for students have been effective in reducing alcohol use; brief interventions that include web-based interactive participation, where normative feedback is provided, also have had positive impacts on alcohol use and other alcohol-related outcomes. Many of these interventions are aimed at helping participants narrow the difference between perceived and actual behaviors of others (i.e., “correcting” the overestimation of use by others) [1,20]. In a recent test of the theoretical underpinnings of social norms theory, Johnson [21] showed that individuals whose perceptions of normative alcohol use became more accurate drank less alcohol.

Most of the substance use perceptions research has been conducted among students and focused on alcohol use. As such, there is a lack of knowledge on perception of tobacco use and cannabis use by others. Determining how frequently overestimations occur in the general population and how perceptions of use by others are associated with substance use (not limited to alcohol use) will yield information about perceptions as a potential factor to target in preventive interventions. Since both substance use and perceptions may be influenced by the behavior of close peers or family members, it is important to take into account whether or not individuals have been exposed to a heavy substance use environment [22].

Therefore, we studied the association between perceptions of tobacco, cannabis, and alcohol use by others with participants’ own tobacco, cannabis, and alcohol use among 20-year-old Swiss men in the general population. We hypothesized that misperceptions of substance use by others are frequent and are associated with participants’ current use.

## Methods

The present study was part of the Cohort Study on Substance Use Risk Factors (C-SURF). Young Swiss men were approached for enrollment in this large cohort study when they presented at army recruitment centers in the French and German sectors of Switzerland, a country that has a mandatory two-day procedure to assess eligibility for military service. Virtually all 20-year-old Swiss men have to participate; thus, C-SURF participants were approached and recruited as they attended the centers at Lausanne (French sector) and Windisch (German sector). Even though Swiss men are approached for enrollment at the same age, some can attend the recruitment centers sooner than others: this explains the age range observed in studies conducted at the army recruitment centers by our group [13,23,24]. Switzerland is made up of 26 cantons (political subdivisions) and half-cantons. The Lausanne center processes conscripts from all of the French-speaking cantons (Berne [French-speaking parts], Fribourg, Genève, Jura, Neuchâtel, Valais, and Vaud), while the Windisch center processes conscripts from eight German-speaking cantons or half-cantons (Argau, Basel-Landschaft, Basel-Stadt, Luzern, Nidwalden, Obwalden, Solothurn, and Uri).

To minimize the risk of under- or over-reporting, participants were informed that all information they provided was confidential and had no implications for army conscription procedures. Participants were notified that the research was not connected to the army, and that military personnel could not see the responses or other data from any individual. Virtually all center attendees were eligible to participate in the study if they gave written informed consent. The Ethics Committee for Clinical Research at the Lausanne University Medical School approved the project.

During the C-SURF enrollment period (August 23, 2010 to July 31, 2011), 12,564 conscripts attended the designated army centers and were given the option of completing a brief self-administered screening questionnaire assessing alcohol and other drug use; 11,819 (94%) completed it (considered hereafter as *census data*). All attendees were offered participation in the cohort study. Within two weeks after enrolment, attendees who gave consent were invited by mail or email to complete the cohort study questionnaire. Cohort study participants (n = 5216) completed a paper-and-pencil or online questionnaire containing items about substance use by others, which asked them to estimate the prevalence of peer tobacco and cannabis use and the percentage of peers who drink more alcohol than they do.

### Cohort study assessment

#### Substance use

The study questionnaire contained questions on drinking frequency (*How often do you have a drink containing alcohol?)* with answer choices of number of days per week (open-ended), 2–3 times a month, monthly or less, or never; and on alcohol quantity (*How many drinks containing alcohol do you have on a typical day when you are drinking?)* with a single open-ended answer (number of standard drinks). The time frame was the past 12 months. Number of standard drinks per week was obtained by multiplying the frequency and quantity questions. A standard drink was defined as 100 ml of wine, 250 ml of beer, 275 ml of pre-mixed drink containing spirits, or 25 ml of spirits (each containing about 10 g ethanol). Pictures of the drink equivalences accompanied each questionnaire.

Tobacco use was assessed with the following items: participants reporting any cigarette use over the past 12 months completed questions on tobacco frequency (*How often, in general, have you smoked cigarettes in the past 12 months?)* with answer choices of every day, 1–2, 3–4, or 5–6 days a week, 2–3 days per month, or once a month or less; and on tobacco quantity (*On a usual day when you smoke cigarettes, how many cigarettes do you smoke?)* with a single open-ended answer (number of cigarettes). The number of cigarettes smoked per week was obtained by multiplying frequency and quantity questions.

Cannabis use was assessed as follows: participants reporting any use by cannabis over the past 12 months completed a question on frequency *(How often have you used cannabis over the past 12 months?)* with answer choices of monthly or less, 2–4 times a month, 2–3 times a week, 4–5 times a week, or every day/almost every day.

#### Perceptions

Perceptions of smoking, cannabis, and alcohol use by others were assessed with the following items: *What is the percentage of men your age that are smoking cigarettes? What is the percentage of men your age that are using cannabis? What is the percentage of men your age who are drinking more alcohol than you do?*

#### Additional variables

Participants were asked whether or not they have family members (parents or siblings) with alcohol or drug problems. Family history was considered positive when at least one parent or sibling had an alcohol or drug problem. Participants were also asked the number of their close friends (none or one, two or some, or most) who have alcohol or drug problems, and were coded positive if they replied with some or most. The three choices were later collapsed into a dichotomous variable (none or one and two or some versus most) to indicate a heavy alcohol or drug use environment. Due to a strong association between presence of close friends with an alcohol problem and presence of close friends with a drug problem (chi-square = 1705.7, 1df, p < 0.0001), those two variables seemed to be carrying the same information; thus, a single variable (presence of close friends with alcohol or drug problems) was created. Education level was also reported.

### Substance use in the census

#### Prevalence of tobacco and cannabis use

Census data from the short screening instrument that 94% of all conscripts completed was used to determine the prevalence of tobacco and cannabis use. To lessen the risk of exaggerated overestimations of prevalence of use in our study, we adopted a conservative attitude towards determining overestimations. Therefore, we chose the most inclusive definition of the prevalence of use in the census: in establishing the prevalence of tobacco and cannabis use, we considered those reporting *any* use of tobacco or cannabis over the past 12 months to be a user.

#### Alcohol consumption

Census norms were computed with the alcohol consumption data from the 11,819 individuals. The drinking-frequency and alcohol-quantity questions were multiplied to obtain the number of standard drinks per week.

### Overestimation, underestimation and accurate estimation of substance use by others

#### Tobacco and cannabis use

Overestimation, underestimation, and accurate estimation of the use of substances by others was determined by assessing how participants rated the prevalence of tobacco and cannabis use among peers of the same age and sex compared with the prevalence established in the census. A perceived prevalence within the ± 10% range of the prevalence established in the census was considered an accurate estimation of use by others. We *a priori* chose a ± 10% range for its clinical utility and to match the definitions found in other studies in the literature [15,25]. A smaller range could lead to a bias in favor of demonstrating that youth overestimate substance use by others. Proportions of conscripts overestimating, underestimating, and accurately estimating the prevalence of tobacco and cannabis use were determined.

#### Alcohol use

Perceived prevalence of alcohol use was not deemed relevant, given the actual high prevalence of alcohol use in the study population [24]. Therefore, estimations of alcohol use were made among alcohol users only, and each participant was asked to estimate how many individuals drank more alcohol than they did [13]. In order to determine for each person the proportion of the census that drank more alcohol than they did, weekly alcohol consumption (in standard drinks) by each of the study participants was compared with the weekly alcohol consumption reported by the census. The proportion of individuals in the census drinking more alcohol than a given study participant was compared with the perceived proportion reported by that participant. The prevalence of overestimation, underestimation, and accurate estimation of peer alcohol drinking was computed in the total sample. An accurate estimation was considered a perceived proportion within the ± 10% range of the computed proportion.

### Association between perception of use by others and substance use

In a first step, separate over-dispersed Poisson regression models for tobacco, cannabis, and alcohol use were created to compare the substance use of those who overestimated, underestimated, and accurately estimated substance use by others. Those accurately estimating the use by others were used as the reference group. The “tobacco” model used the perception of tobacco use by others as the independent variable, the “cannabis” model used the perception of cannabis use by others as the independent variable, and the “alcohol” model used the perception of alcohol use by others as the independent variable. Outcome measures were number of cigarettes smoked per week for the “tobacco” model, number of days with cannabis use per month for the “cannabis” model, and number of standard drinks per week for the “alcohol” model. All three models were adjusted for age, education level, and linguistic sector. In a second step, presence of close friends with alcohol or drug problems and family history of alcohol or drug problems were added to the models, then adjusted for age, education level, and linguistic sector.

## Results

Table 1 shows the characteristics of the 5216 participants. Nearly all (91.8%) of the conscripts reported some alcohol use in the past 12 months; about half (47.5%) used tobacco, and nearly a third (30.9%) used cannabis. Among tobacco smokers, the mean (SD) number of cigarettes per week was 48.1 (54.8). Among cannabis users, the mean number of days per month using cannabis was 7.0 (9.2). For the alcohol users, the mean number of drinks per week was 8.4 (14.0). In the census (n = 11,819), past-12-month prevalence was 44.5% for any tobacco use, 36.3% for any cannabis use, and 90.6% for any alcohol use.

**Table 1:** Participant characteristics

**Participants characteristics (n = 5216)**	
Age, mean (SD)	19.5 (1.3)
German speaking, n (%)	2097 (40.2%)
Elementary education only, n (%)	2523 (48.4%)
Any drinking, past 12 months, n (%)	4789 (91.8%)
Mean number of drinks*/week among drinkers (SD)	8.4 (14.0)
Any tobacco use, past 12 months, n (%)	2478 (47.5%)
Mean # of cigarettes/week among users (SD)	48.1 (54.8)
Any cannabis use, past 12 months, n (%)	1612 (30.9%)
Mean # of days/month with cannabis among users (SD)	7.0 (9.2)
Close friends with alcohol or drug problems,** n (%)	711 (13.6%)
Family history of alcohol problems,*** n (%)	388 (7.4%)
Family history of drug problems,*** n (%)	173 (3.3%)

*Standard drink: 100 ml of wine; 250 ml of beer; 275 ml of premixed drink containing spirits; or 25 ml of spirits (each about 10 g of ethanol).

**When participants reported that “some” or “most” of their close friends (versus “none” or “one or two”) had an alcohol or drug problem, the variable was coded positively.

***We considered that a family history of alcohol or drug problems was present when participants reported at least one parent or sibling with an alcohol or drug problem.

### Perception of substance use by others

The prevalence of tobacco use was overestimated by 46.1% and accurately estimated by 37.3% of study participants. The prevalence of cannabis use was overestimated by 21.8% and accurately estimated by 31.6% of the participants. When comparing their own alcohol use with alcohol use by others, more than half of participants (53.4%) overestimated, while 31.0% accurately estimated. The proportion of participants overestimating, accurately estimating, and underestimating substance use is reported in Table 2.

**Table 2:** Overestimation, accurate estimation, and underestimation of others substance use

	**Overestimation**	**Accurate estimation**	**Underestimation**
Tobacco*	46.1%	37.3%	16.6%
Cannabis*	21.8%	31.6%	46.6%
Alcohol**	53.4%	31.0%	15.6%

*Using census data, we determined whether participants overestimated or underestimated the prevalence of tobacco or cannabis use by 10% or more.

**The proportion of participants in the census drinking more than a given participant in the sample was compared with the perceived proportion reported by each of the participants of the study sample. The prevalence of overestimation, underestimation, and accurate estimation of peer drinking was computed for the total sample. An accurate estimation was considered a perceived proportion within the ± 10% range of the computed proportion.

Participants overestimating, accurately estimating, and underestimating tobacco use by others reported a mean (SD) of 25.4 (47.6), 20.9 (42.3), and 20.2 (41.8) cigarettes smoked per week, respectively. Participants overestimating, accurately estimating, and underestimating cannabis use by others reported a mean (SD) of 3.8 (7.9), 2.3 (6.2), and 1.3 (4.5) days of cannabis use per month, respectively. Participants overestimating, accurately estimating, and underestimating alcohol use by others reported a mean (SD) of 11.1 (16.0), 7.0 (12.4), and 2.9 (2.2) drinks per week, respectively.

### Associations between perception of use by others and substance use

Separate over-dispersed Poisson regression models for tobacco, cannabis, and alcohol use were used to assess the associations between perception of use by others and substance use. All models were adjusted for age, education level, and linguistic sector. Results are expressed as incidence rate ratios (IRR) for the count variable in the model. The three count variables under study were number cigarette smoked per week, number of day of cannabis use per month, and number of standard alcohol drinks per week. The IRR gives the factor change in the expected count compared with the reference category. For example, if participants with characteristic X are compared with the reference group without the characteristic X with respect to their weekly alcohol use, an IRR = 1 means that, holding all other variables constant, having the characteristic X does not change the expected number of drinks per week. An IRR = 0.8 means that, on average, the group with the characteristic X reports 20% less drinks per week than the group without the characteristic X and an IRR = 2 means that, on average, the group with the characteristic X reports twice the number of drinks per week than the group without the characteristic X.

#### Tobacco use

Compared with participants accurately estimating tobacco use by others, those who overestimated it reported more cigarettes smoked per week (incidence rate ratio (IRR) [95% CI], 1.19 [range, 1.06, 1.34]). There was no difference in the number of cigarettes smoked per week between those underestimating and those accurately estimating tobacco use by others (IRR [95% CI], 0.97 [range, 0.82, 1.15]).

#### Cannabis use

Compared with participants accurately estimating cannabis use by others, those who overestimated it reported more days of cannabis use per month (IRR [95% CI], 1.59 [range, 1.33, 1.89]), whereas those who underestimated it reported less days of cannabis use per month (IRR [95% CI], 0.57 [range, 0.47, 0.69]).

#### Alcohol use

Compared with participants accurately estimating alcohol use by others, those who overestimated it reported consuming more drinks per week (IRR [95% CI], 1.57 [range, 1.42, 1.74]), whereas those who underestimated it reported consuming fewer drinks per week (IRR [95% CI], 0.41 [range, 0.33, 0.50]).

Table 3 shows the results of the same three models after adding presence of close friends with an alcohol or drug problem, family history of alcohol problems, and family history of drug problems. Independent associations of perceptions with substance use remained. Compared with participants accurately estimating tobacco use by others, those who overestimated it reported more cigarettes smoked per week (IRR [95% CI], 1.17 [range, 1.05, 1.32]). There was no difference in the number of cigarettes smoked per week between those underestimating and those accurately estimating tobacco use by others (IRR [95% CI], 0.99 [range, 0.84, 1.17]). Compared with participants accurately estimating cannabis use by others, those who overestimated it reported more days of cannabis use per month (IRR [95% CI], 1.43 [range, 1.21, 1.70]), while those who underestimated it reported fewer days of cannabis use per month (IRR [95% CI], 0.62 [range, 0.23, 0.75]). Compared with participants accurately estimating alcohol use by others, those who overestimated it reported more drinks consumed per week (IRR [95% CI], 1.57 [range, 1.43, 1.72]), while those who underestimated it reported fewer drinks consumed per week (IRR [95% CI], 0.41 [range, 0.34, 0.50]). There were the following significant positive associations: having close friends with alcohol or drug problems and one’s own tobacco, cannabis, and/or alcohol use; family history of alcohol problems and one’s own tobacco and/or alcohol use; and family history of drug problems and one’s own tobacco, alcohol, and/or cannabis use.

**Table 3:** Multivariable models examining the association of substance use perception, presence of close friends with alcohol or drug problems, and family history of alcohol or drug problems with substance use

Model 1: tobacco use*	**Cigarettes/week, IRR (95% CI)**
*Perception of tobacco use of others (same age/sex), reference group = accurate estimation of tobacco use of others*	
Overestimation of tobacco use by others	1.17 (1.05; 1.32)
Underestimation of tobacco use by others	0.99 (0.84; 1.17)
Close friends with alcohol or drug problems	2.08 (1.83; 2.35)
Family history of alcohol problems (parents/siblings)	1.48 (1.25; 1.75)
Family history of drug problems (parents/siblings)	1.52 (1.22; 1.89)
Model 2: cannabis use*	**Days of cannabis use/month, IRR (95% CI)**
*Perception of cannabis use of others (same age/sex), reference group = accurate estimation of cannabis use of others*	
Overestimation of cannabis use by others	1.43 (1.21; 1.70)
Underestimation of cannabis use by others	0.62 (0.23; 0.75)
Close friends with alcohol or drug problems	2.91 (2.48; 3.42)
Family history of alcohol problems (parents/siblings)	1.12 (0.89; 1.42)
Family history of drug problems (parents/siblings)	1.78 (1.36; 2.33)
Model 3: drinking*	**Drinks/week, IRR (95% CI)**
*Perception of drinking of others (same age/sex), reference group = accurate estimation of drinking of others*	
Overestimation of drinking of others	1.57 (1.43; 1.72)
Underestimation of drinking of others	0.41 (0.34; 0.50)
Close friends with alcohol or drug problems	1.43 (1.28; 1.59)
Family history of alcohol problems (parents/siblings)	1.35 (1.17; 1.55)
Family history of drug problems (parents/siblings)	1.38 (1.14; 1.67)

*All models were adjusted for age, education level, and linguistic region. IRR = incidence rate ratio.

## Discussion

Perceptions of substance use by others are associated with one’s own use among young men; specifically, our results show that overestimating substance use by others is associated with greater consumption. In addition, underestimating the substance use by others appears associated with less use, except for tobacco. Our study adds important information about the frequency of overestimation, underestimation, and accurate estimation of substance use and the association of overestimation of use by others with current use, especially for tobacco and cannabis use where evidence has been scarce [2,16,26].

The magnitude of the associations between perceptions and usage was similar in the models where variables, such as having close friends with alcohol or drug problems or having a family history of alcohol or drug problems, are added or taken out. The relationship most affected by the addition of these variables in multivariable models was overestimation of cannabis use by others and participants’ own cannabis use (IRR 1.59 versus 1.43 in the model containing the close friends with alcohol or drug problems and family history variables). These results are in line with the literature [11,13,14,16]. The magnitude of the observed associations between perceptions of use by others and substance use are of clinical significance: compared with participants accurately estimating alcohol use by others, participants who overestimated alcohol use by others reported drinking almost 60% more per week. Those underestimating alcohol use by others reported drinking 60% less per week than those accurately estimating. For cannabis, these differences were also of clinical significance and were in the 40% range. Though smaller, the association between perception of tobacco use by others and tobacco use was still clinically significant: participants overestimating tobacco use by others smoked 17% more cigarettes per week compared with those accurately estimating it.

As a potential target of normative feedback interventions, overestimations of use by others have been the focus of various research studies. Our results show that overestimations of substance use by others are frequent among young men (but vary by substance) and are associated with greater consumption. These associations are independent of being in a heavy alcohol or drug using environment, indicating that, even in the case of proximity to these groups, overestimations of use by others may influence one’s own use. Our results show that the overestimation of use by others has a stronger association with one’s own use for cannabis and alcohol than it does for tobacco. This suggests a potentially differential influence of perceptions on one’s own use of these substances. An alternate explanation for overestimations is that individuals select their friends and acquaintances based on shared preferences in alcohol, tobacco, and drug use, and overestimations of behaviors within a “distal” group of the same of peers of same age and sex may reflect peer usage more than anything else may. Using “distal” groups in research on substance use perceptions (i.e., individuals of the same age and gender as opposed to more “proximal” groups such as friends or people in the same fraternity/sorority) has faced criticism and has been presented as a source of exaggerated overestimations [27]. We attempted to take into account the influence of a heavy using environment and heritability by showing independent associations of one’s own use and overestimations of others’ use with presence of close friends and family members with alcohol and drug problems. Even though misperceptions of a distal group are less likely to have an influence on behavior than misperceptions of a proximal group, we still observed associations between overestimations and substance use [28].

We observed strong variations in the frequency of overestimation by substance: overestimation of alcohol and tobacco use was observed in more than 45% of the participants, while overestimation of cannabis use was observed in 22% of participants. We do not have a straightforward explanation for the lower rates of overestimation of cannabis use. One possible explanation is that cannabis may have received less attention than tobacco and alcohol, or its consumption may be less visible due to its illegal status in Switzerland. Determining the causes of these variations is an area for future research.

The association of secondary variables (close friends with alcohol or drug problems, and family substance use history) with participants’ own substance use is concordant with research showing that environmental [29-31] and genetic influences [32] play a role in individual’s substance use [22]. In the present study, both family history of drug or alcohol problems and markers of peer behavior were associated with substance use. Family history can be seen both as a marker of genetic and environmental influences, since heritability and parental and family-member modeling of substance use behaviors can be associated with substance use [22,32,33]. Generally, family history associations were found across substances: family history of drug problems was associated with alcohol, tobacco, and cannabis use, and family history of alcohol problems was associated with alcohol and tobacco use. The presence of close friends with alcohol or drug problems (a proxy for a heavy using environment) was also associated with substance use.

This study has several limitations. First, it should be noted that we relied on self-reported measures for substance use and other factors. Notably, participants were asked to report whether or not they had family members or friends with alcohol and or drug problems, and the psychometric properties of these questions were not tested. Second, we assessed only descriptive norms, and interpersonal perception was limited to those of the same age and sex as the participant. Perception of the amount of alcohol used by others in more proximal groups (such as close friends) was not evaluated. The large number of participants in the study and the logistics of the screening survey preclude gathering detailed data that could be obtained during face-to-face structured interviews. In addition, we *a priori* chose a ± 10% range to define “accurate perception.” A smaller range could have introduced a bias towards demonstrating that youth overestimate substance use by others; however, it should be noted that this definition impacts the prevalence of over-, under-, and accurate estimations. Sensitivity analyses around this criterion were performed (using a ± 5% and a ± 20% range). We did not observe major differences in the associations reported herein. Third, because it is a cross-sectional design, we were not able to investigate possible causal pathways or assess the influence of changes in perception on substance use. In addition, only 20-year-old men were included, and our results cannot be generalized beyond this population subset. Evidence suggests that the magnitude of norm misperception is influenced by gender. Even though misperceptions have been observed across gender, their magnitude is likely to differ. Therefore, our results should not be extrapolated to women. Fourth, about 6% of the invited conscripts refused to complete the screening questionnaire; therefore, we cannot be certain that alcohol, tobacco, and cannabis use was accurately measured in the census. Nevertheless, it is unlikely that this unduly affected measures of substance use prevalence in the census or significantly influenced the obtained perceptions, since we were able to rely on screening data from 94% of the census. Cohort participation and response to cohort questionnaires may be subject to self-selection bias. Although minimally, participants who did complete the cohort questionnaire differed from the source population. Substance use was lower among those who completed the cohort questionnaire compared with census data. The non-response bias was <10% for alcohol and cannabis and <15% for tobacco use. Detailed analyses are presented elsewhere [34,35].

In 2012, Hilde Pape pointed out methodological limitations in studies focusing on estimations of peer substance use and argued that these restrictions may lead to exaggerated results [27]. She reported that the representativeness of studies in the field is problematic because of convenience samples and low response rates. In this regard, our study has the advantage of using a large sample with perception items imbedded within a larger cohort questionnaire. Also, because 94% of the source population was assessed on substance use, the representativeness of our cohorts has been well characterized. Even if differences existed between cohort participants and the source population, these differences were limited [34]. Other methodological concerns reported by Pape include the complexity of questions used to assess perceptions. Particularly for the item on alcohol use perception, this limitation exists in the present study. Asking participants to compare their alcohol use to alcohol use by others by having them determine the proportion of individuals their age and sex who drink more alcohol than they do can be challenging, and assumes pre-existing beliefs about the use of others. Questions on tobacco and cannabis use perceptions were less complex, even though they also assumed pre-existing beliefs and did not include “I don’t know” as a choice. There is no way to determine the level or strength of pre-existing beliefs in an individual. Therefore, the present study is also limited by the complexity of the questions used to assess perceptions.

Nevertheless, we believe our study has some notable strengths. We were able to assess actual and perceived substance use in samples from the same population that included consumption screening data from 94% of the census and from a large sample of cohorts. We took into account proxies of peer use in the adjusted analyses, which allowed an assessment of independent associations between perceptions of use and one’s own use. Even though interpersonal perceptions were limited to peers of the same age and sex, the fact that we assessed family history and the presence of close friends with substance use problems strongly supports the hypothesis of an independent association of perceived substance use by others with one’s current own use. Also, we used norms that are usually used in available normative feedback interventions (i.e., in currently available interventions, notably web-based normative-feedback interventions, substance use by a given individual is compared with the substance use of a population of the same age and sex, and not to the consumption of close friends, which would be challenging to determine).

## Conclusion

Our results confirm among young men in the general population what has already been seen in selected student populations; i.e., overestimations of substance use occur frequently and are associated with greater usage. Although we cannot make a causal interpretation because of our study design, our results suggest that perceptions themselves may influence current behavior, and that those who overestimate substance use by others are indeed likely to use more themselves. We believe our study adds information on the prevalence of misperceptions for substances other than alcohol. It is important to note that 30-40% of the participants accurately estimated use by others, and that a substantial proportion underestimated use by others.

If preventive interventions are to be based on normative feedback and their aim is to reduce misperceptions, then the prevalence of overestimation indicates that they may benefit about half of the population, or in the case of cannabis, as little as about 20% of the population. To be successful, such interventions should take into account differential strengths of association across substances, especially if large-scale efforts to implement interventions based primarily on this theoretical rationale are attempted.

## Competing interests

The authors declare that they have no competing interests.

## Authors’ contributions

NB designed the study, analyzed the data, and wrote the initial draft and final version of the article. JS participated in planning and data analyses, discussed earlier versions, and reviewed the final version of the article. MF participated in planning and data analyses, reviewed the analyses, discussed earlier versions, and reviewed the final version of the article. J-BD designed the study discussed earlier versions and reviewed the final version of the article. GG designed the study, discussed analyses, discussed earlier versions and reviewed the final version of the article. All authors contributed to and approved the final manuscript.
